# An external validation of the Candiolo nomogram in a cohort of prostate cancer patients treated by external‐beam radiotherapy

**DOI:** 10.1186/s13014-021-01814-5

**Published:** 2021-05-05

**Authors:** Domenico Gabriele, Alessia Guarneri, Sara Bartoncini, Fernando Munoz, Matteo Tamponi, Filippo Russo, Georgios Stamatakos, Caterina Guiot, Daniele Regge, Umberto Ricardi

**Affiliations:** 1 Department of Radiology, Città della Salute e della Scienza Hospital, University of Torino, via Genova 3, 10126 Turin, Italy; 2 Department of Radiology, FPO-IRCCS Candiolo Cancer Institute, Candiolo, Italy; 3 Department of Radiation Oncology, University of Torino, Città della Salute e della Scienza Hospital, Turin, Italy; 4 Department of Radiation Oncology, Regional Hospital of Aosta, Aosta, Italy; 5 Sardinian Regional Health Service, Sassari, Italy; 6 Institute of Communication and Computer Systems, National Technical University of Athens, Athens, Greece; 7 Department of Neuroscience, University of Torino, Turin, Italy

**Keywords:** Prostate cancer, Radiotherapy, Nomogram, External validation

## Abstract

**Background:**

the aim of this study is to perform an external validation for the Candiolo nomogram, a predictive algorithm of biochemical and clinical recurrences in prostate cancer patients treated by radical Radiotherapy, published in 2016 on the journal “Radiation Oncology”.

**Methods:**

561 patients, treated by Radiotherapy with curative intent between 2003 and 2012, were classified according to the five risk-classes of the Candiolo nomogram and the three risk-classes of the D’Amico classification for comparison. Patients were treated with a mean prostatic dose of 77.7 Gy and a combined treatment with Androgen-Deprivation-Therapy in 76% of cases. The end-points of the study were biochemical-progression-free-survival (bPFS) and clinical-Progression-Free-Survival (cPFS). With a median follow-up of 50 months, 56 patients (10%) had a biochemical relapse, and 30 patients (5.4%) a clinical progression. The cases were divided according to D’Amico in low-risk 21%, intermediate 40%, high-risk 39%; according to Candiolo very-low-risk 24%, low 37%, intermediate 24%, high 10%, very-high-risk 5%. Statistically, the Kaplan-Meier survival curves were processed and compared using Log-Rank tests and Harrell-C concordance index.

**Results:**

The 5-year bPFS for the Candiolo risk-classes range between 98 and 38%, and the 5-year cPFS between 98 and 50% for very-low and very-high-risk, respectively. The Candiolo nomogram is highly significant for the stratification of both bPFS and cPFS (P < 0.0001), as well as the D’Amico classification (P = 0.004 and P = 0.001, respectively). For the Candiolo nomogram, the C indexes for bPFS and cPFS are 75 and 80%, respectively, while for D’Amico classification they are 64 and 69%, respectively. The Candiolo nomogram can identify a greater number of patients with low and very-low-risk prostate cancer (61% versus 21% according to D’Amico) and it better picks out patients with high and very-high-risk of recurrence, equal to only 15% of the total cases but subject to 48% (27/56) of biochemical relapses and 63% (19/30) of clinical progressions.

**Conclusions:**

the external validation of the Candiolo nomogram was overall successful with C indexes approximately 10% higher than the D’Amico control classification for bPFS and cPFS. Therefore, its clinical use is justified in prostate cancer patients before radical Radiotherapy.

*Trial registration* Retrospectively registered.

## Background

Prostate cancer is the most common cancer in men and the second leading cause of cancer death in males [1]. The early prediction of prostate cancer recurrences has inspired several modeling approaches, from classical statistical algorithms [2] till to more complex artificial intelligence methods, among which nomograms are very practical and popular tools. A lot of nomograms have been developed to guide therapy and predict outcomes after radical Radiotherapy: one of the most popular classifications are D’Amico’s risk classes, which divide patients by pre-treatment PSA, clinical stage and biopsy Gleason Score (bGS) in three categories: low risk (PSA < 10 ng/ml and cT1–cT2a and bGS ≤ 6), intermediate risk (PSA 10–20 ng/ml or cT2b or bGS 7) and high risk (PSA > 20 ng/mL or clinical stage ≥ cT2c or bGS ≥ 8) [3, 4].

A new classification tool, the Candiolo nomogram (co-funded by the European Commission through the CHIC project, “Computational Horizons In Cancer: Developing Meta and Hyper-Multiscale Models and Repositories for In-Silico Oncology”, Grant Agreement 600841), was published in 2016 on the journal Radiation Oncology [5]. It predicts the risk of biochemical recurrence from prostate cancer in patients undergoing radical radiotherapy by dividing patients into five risk classes by combining five pre-treatment parameters, i.e. age, PSA at diagnosis, clinical-radiological staging, biopsy Gleason Score (bGS) and percentage of biopsy positive cores (%PC).

**Fig. 1:**
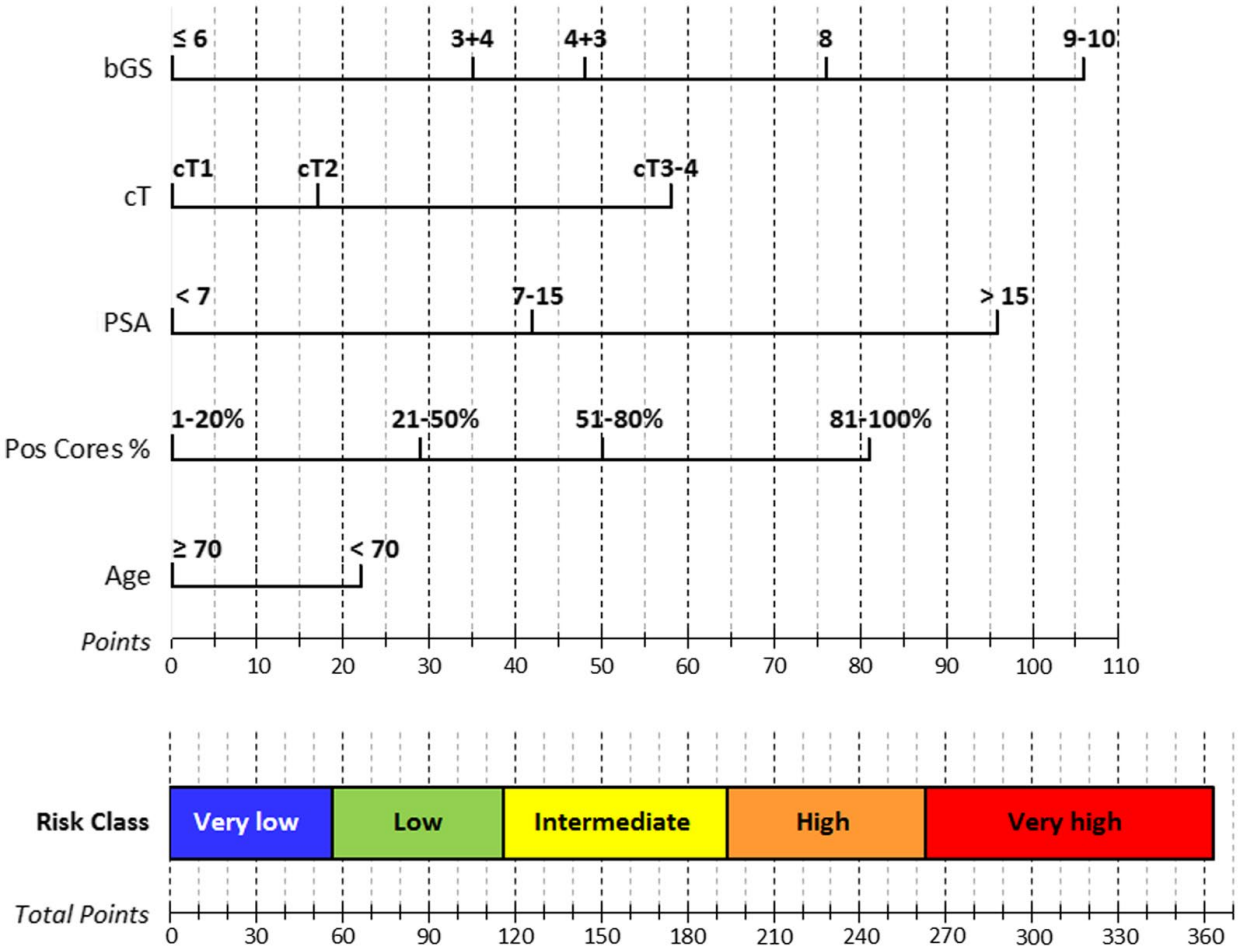
The Candiolo nomogram

In particular, the five parameters were categorized as follows: age ≥ 70 years or age < 70 years; PSA < 7 ng/mL, 7–15 ng/mL or > 15 ng/mL; clinical-radiological stage cT1, cT2 or cT3-cT4; bGS ≤ 6, 3 + 4, 4 + 3, 8 or 9–10; %PC 1–20%, 21–50%, 51–80% or 81–100%. Then, the patients were split into five risk classes (very-low, low, intermediate, high, and very-high) according to the Candiolo nomogram scores shown in Fig. 1 and Table 1.

**Table 1:** Candiolo nomogram’s scores

bGS	≤ 6	3 + 4	4 + 3	8	9–10
Points	0	35	48	76	106
cT	cT1	cT2	cT3-4		
Points	0	17	58		
PSA	< 7	7–15	> 15		
Points	0	42	96		
%PC	1–20%	21–50%	51–80%	81–100%	
Points	0	29	50	81	
Age	≥ 70 yy	< 70 yy			
Points	0	22			
Risk-class	Very-low	Low	Intermediate	High	Very-high
Total points	0–56	57–116	117–193	194–262	263–363

The nomogram training cohort consisted of 2493 men belonging to the EUREKA-2 multicenter retrospective database on prostate cancer; they were treated with external-beam radiotherapy (EBRT) as primary treatment in north-western Italy between 1997 and 2012. The reproducibility of the multivariate analysis Cox regression model was verified with a bootstrap statistic as internal validation. The Candiolo nomogram was highly significant for the prediction of biochemical Progression Free Survival (bPFS) overall (log-rank test with P < 0.0001) and for the distinction of paired curves (all log-rank tests for paired curves with P < 0.001). The nomogram was then applied to the secondary end-points clinical Progression Free Survival (cPFS), systemic Progression Free Survival and Prostate Cancer Specific Survival (overall log-rank tests always with P < 0.0001).

Thereafter, the Candiolo nomogram was compared with the classification of D’Amico made up of three risk classes. The Candiolo nomogram in the training cohort exceeded the D’Amico classification for all the outcomes considered with Harrell’s C Concordance Indexes of 71.5% versus 63% for bPFS and 75.5% versus 65.5% for cPFS. For the five risk classes of the Candiolo nomogram the five-year bPFS were 94%, 85%, 80%, 67 and 43%, while for the three D’Amico risk classes they were much more compressed at 91%, 83 and 72%. Similarly, the cPFS for the Candiolo nomogram were 97%, 94%, 92%, 79 and 62%, while for the D’Amico classes they were 96%, 91 and 85%.

The main limitation of the Candiolo nomogram study is the lack of an external validation. This study aims to verify the reliability of the Candiolo nomogram on an independent database of patients treated by EBRT, always using the D’Amico classification as a control.

## Methods

 The Department of Radiation Oncology of “Città della Salute e della Scienza” hospital, University of Torino, provided the validation cohort. The validation study proposed by Prof. U. Ricardi was approved by the hospital Ethics Committee on the 25th September 2015 as a retrospective historical cohort study on prostate cancer patients treated by radical EBRT.

The validation database was recorded in Excel format (®Microsoft Corporation, Redmond, Washington, USA) and included diagnostic data, comprising the data necessary for the attribution of the risk class of the Candiolo nomogram (age, pre-treatment PSA, clinical-radiological staging, bGS, number of total and positive biopsy cores), therapeutic data on the performed Radiotherapy and Androgen Deprivation Therapy (ADT), and biochemical and clinical follow-up of the patients.

The inclusion criteria of the study were: histological diagnosis of prostate adenocarcinoma; radical Radiotherapy as a first-line treatment, performed with conformational or intensity-modulated technique; temporal consecutiveness of the clinical cases collected. Two Radiation Oncologists, A. Guarneri and S. Bartoncini, collected the clinical data of 930 patients treated consecutively in their Department between 1st January 2003 and 31st December 2012.

In all patients, staging included medical history, physical examination with Digital Rectal Examination (DRE), serum PSA, and Trans-Rectal Ultrasound-guided biopsy of the prostate (TRUS) with histological evaluation of the biopsy Gleason Score (bGS). Radiological exams (abdominal CT, endo-rectal or pelvic MRI and bone scan) were performed according to the patient’s risk class and the opinion of the referring physician. Pre-treatment PSA was dosed prior to biopsy and radiological studies; in case of multiple pretreatment PSA exams, the highest PSA (zenith PSA) was recorded. Primary, secondary, and total bGS were attributed according to the 2005 ISUP Gleason Score review system [6]. The clinical-radiological stage of the primary tumor cT was obtained according to the 2011 AJCC 7th edition staging system [7] by integrating the clinical examination with all available radiological information, while data on the extent of cancer at biopsy were not taken into consideration.

All the patients were treated with Conformational Radiotherapy (3DCRT) or Intensity Modulated and Image-Guided Radiotherapy (IMRT-IGRT) with a curative intent. Fractionation schedules for the prostate CTV (Clinical Target Volume of the prostate) varied between standard fractionation of 2 Gy per fraction and moderate hypo-fractionation of 2.7 Gy per fraction. The treatment scheme consisted of either exclusive Radiotherapy or combined Radiotherapy and Androgen Deprivation Therapy (ADT). The androgen deprivation drugs used were anti-androgens or LHRH-analogues or TAB (Total Androgenic Block, i.e. the combination of the two previous drugs).

Standard follow-up included PSA and DRE every 3 months for the first 2 years, every 6 months until the fifth year, and annually thereafter.

The end-points considered were the biochemical Progression Free Survival (bPFS) and the clinical Progression Free Survival (cPFS). Biochemical recurrence was assessed according to the definition of the Phoenix consensus conference [i.e. an increase of 2 ng/mL or greater compared to post-irradiation nadir PSA [8]]. Clinical relapse was defined as a recurrence in the irradiated prostate gland, or in the regional pelvic lymph nodes or as distant metastases demonstrated by radiological exams (bone scan, choline-PET-CT, MRI, CT, ultrasound), or by a clinical examination, or by biopsy.

Regarding the privacy of patients’ personal data, a pseudo-anonymization procedure was performed, i.e. only the clinical data, and not the personal data, were sent outside the hospital in the database for data analyses.

Statistical analyses were performed by D. Gabriele and M. Tamponi using the statistical software Stata SE 14.0 (®StataCorp, Texas, USA).

The data were filtered to be complete for all the diagnostic parameters mandatory for the application of the Candiolo nomogram. This procedure led to a loss of 369 patients (354 without the number of positive and total biopsy cores, 10 without bGS, 3 without PSA, 2 without staging) leading to a reduction of the validation cohort from 930 to 561 patients.

The %PC was calculated by multiplying 100 by the number of prostate cancer positive cores, of any length, and then dividing by the total number of cores sampled. Age at treatment was calculated as the difference in years between the first day of radiotherapy and the date of birth. The follow-up time was calculated as the difference in months between the date of the patient’s last follow-up and the last day of radiotherapy, rounded to the nearest whole number. The categorical variables were coded in numerical format, both as ordinal variables (for example 0,1,2,3, etc.) and as dummy variables with reference cell coding system (0, 1).

All radiotherapy doses were normalized to an Equivalent Dose at 2 Gy per fraction (ED2Gy) using a mean α/β ratio of 2.5 Gy for prostate cancer (according to the literature the α/β for prostate cancer varies between 1.5 and 5.7 Gy [9–11]).

Table 2 presents the main clinical-epidemiological data of the 561 patients under analysis. The median follow-up was 50 months. During the follow-up 56 patients (10% of the total) had a biochemical recurrence and 30 (5.4%) had a clinical-radiological progression (10 cases relapsed in the prostate, 9 in the pelvic lymph nodes and 18 had bone metastases).

**Table 2:** Clinical-epidemiological features of our validation series of 561 patients

Clinical characteristics	
*Follow-up, mo*	
Mean (SD)	56.5 (27.7)
Median (min–max)	50 (3–146)
*Age, yy*	
Mean (SD)	71.9 (5.7)
Median (min–max)	73 (51–88)
*PSA, ng/mL*	
Mean (SD)	12.93 (30.96)
Median (min–max)	7.70 (1.14–680)
*T staging, no (%)*	
cT1	355 (63%)
cT2	182 (33%)
cT3-4	24 (4%)
*bGS, no (%)*	
≤ 6	220 (39%)
3 + 4	174 (31%)
4 + 3	69 (12%)
8	55 (10%)
9–10	43 (8%)
*Biopsy cores sampled, no*	
Mean (SD)	11.0 (4.6)
Median (min–max)	10 (2–35)
*%PC, %*	
Mean (SD)	41.3% (27.8)
Median (min–max)	38% (4–100)
*N staging, %*	
Not performed	72%
performed N0	27.3%
performed N1	0.7%
*M staging, %*	
Not performed	74%
performed M0	26%
*D’Amico risk class, no (%)*	
Low	119 (21%)
Intermediate	223 (40%)
High	219 (39%)
*Candiolo risk class, no (%)*	
Very-low	133 (24%)
Low	211 (37%)
Intermediate	133 (24%)
High	56 (10%)
Very-high	28 (5%)
*RT dose to prostate-CTV, ED2Gy, α/β* = *2,5*	
Mean (SD)	77.7 (2.4)
Median (min–max)	78 (72–82)
*Fractionation schedule, %*	
Std fractionation 2 Gy /fr	77%
Hypo-fractionation 2,7 Gy /fr	23%
*RT technique, %*	
3DCRT	77%
IMRT-IGRT	23%
*Seminal vesicles irradiation, %*	
No	22%
Yes	78%
*Pelvic nodal irradiation, %*	
No	98%
Yes	2%
Exclusive RT	24%
RT + ADT	76%
*ADT duration, mo*	
Mean (SD)	13.0 (10.1)
Median (min–max)	8 (1–46)
*ADT drug, %*	
Anti-Androgen	37%
LHRH-analogue	49%
TAB	14%

SD, standard deviation; ED2Gy, equivalent dose at standard dose of 2 Gy per fraction; RT, radiotherapy; ADT, androgen deprivation therapy

Mean age was 71.9 years, mean PSA 12.93 ng/mL, 63% of patients were staged cT1, 32% cT2 and only 4% cT3 or cT4, 43% had a bGS of 7, followed by 39% with bGS ≤ 6 and 18% with a bGS ≥ 8. The number of biopsy cores sampled was on average 11, with a mean percentage of biopsy positive cores of 41.3%. Only 4 patients (0.7%) were classified as cN1 at staging.

According to the D’Amico classification, patients were at low, intermediate, and high risk in 21%, 40 and 39% of cases, respectively. According to the Candiolo nomogram, patients were at very-low, low, intermediate, high, and very-high risk in 24%, 37%, 24%, 10 and 5% of cases, respectively.

The mean RT dose to the prostate CTV was 77.7 Gy. The fractionation schedule was standard at 2 Gy/fraction with 3DCRT technique in 77% of cases and moderately hypo-fractionated at 2.7 Gy/fraction with IMRT-IGRT technique in 23% of cases. Seminal vesicles were irradiated in 78% of patients, while pelvic lymph nodes only in 2% of cases. Treatment consisted of exclusive Radiotherapy or Radiotherapy combined with ADT in 24 and 76% of cases, respectively. When administered, the ADT had a median duration of 8 months (and a mean of 13 months) and the drugs used were anti-androgens in 37% of patients, LHRH-analogues in 49% or TAB in 14% of cases.

The 561 patients were then assigned to the risk classes of the Candiolo nomogram according to the scores described in Table 1, and the patients were also categorized into the three risk classes of the D’Amico classification for comparison.

The Kaplan-Meier survival curves for bPFS and cPFS were graphed for the two classifications of Candiolo and D’Amico. The statistical significance for the whole set of curves and for couples of curves were calculated using Log-Rank tests.

The Harrell C concordance index was also calculated to evaluate the overall accuracy and predictive ability of the classifications. The concordance index was calculated according to the formula C = (E + T/2) /P, where P are the survival comparison Pairs combined among the N subjects analyzed, E are the number of pairs ordered as Expected and T the number of non-informative predictions (Tied pairs).

## Results

**Fig. 2:**
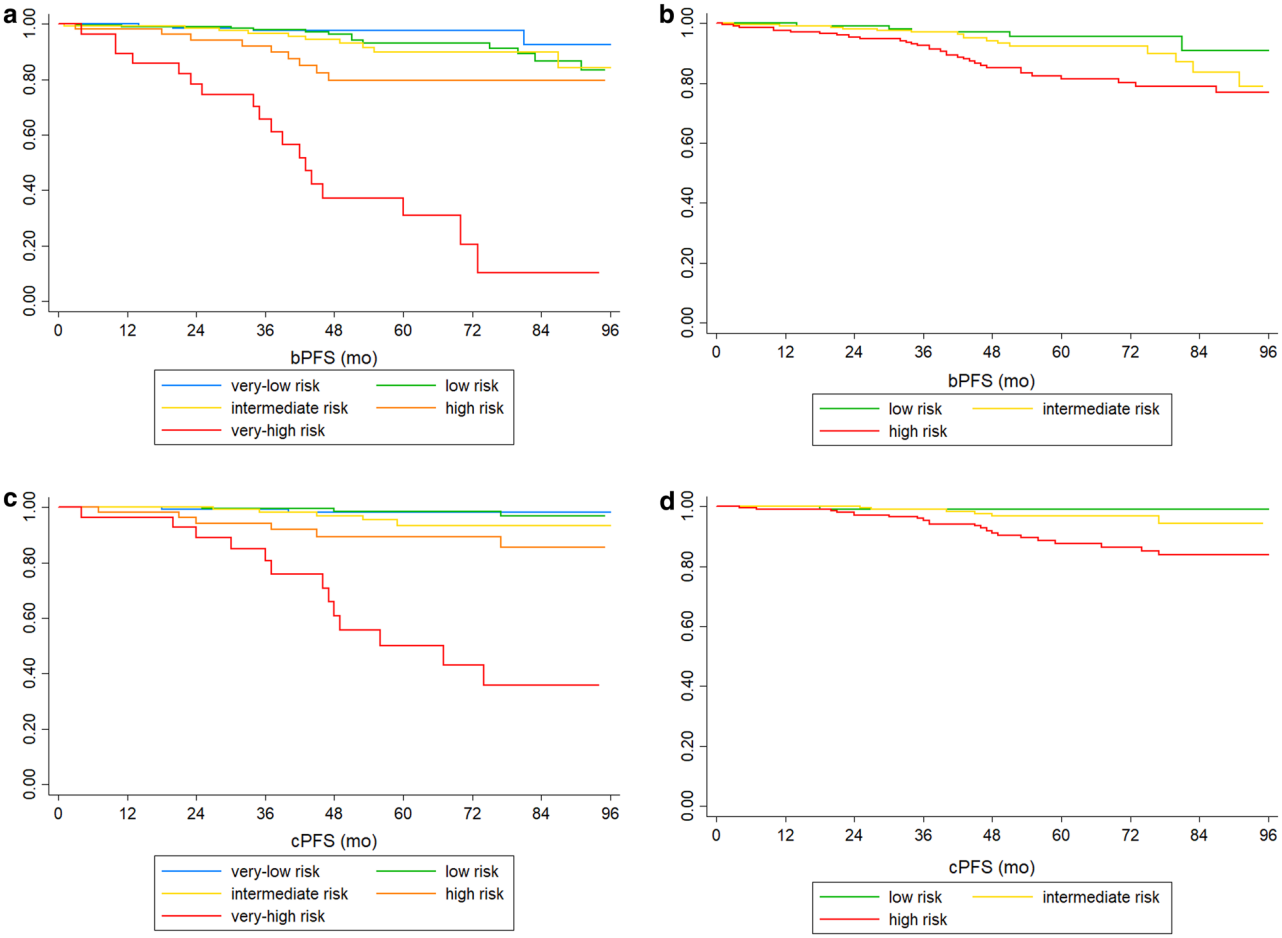
Validation cohort bPFS (**a**, **b**) and cPFS (**c**, **d**) for Candiolo nomogram (**a**–**c**) and D’Amico classification (**b**–**d**)

Kaplan–Meier survival curves for bPFS and cPFS for the five risk classes of the Candiolo nomogram and for the three risk classes of D’Amico classification are shown in Fig. 2.

For the Candiolo nomogram, the 5-year bPFS range between 98%, 93%, 90%, 80 and 38% for very-low, low, intermediate, high, and very-high risk, respectively, as well as 5-year cPFS vary between 98%, 98%, 94%, 89 and 50%, respectively. For the three classes of D’Amico classification, the 5-year bPFS vary between 95%, 92 and 83% for low, intermediate, and high risk, respectively, while the 5-year cPFS range between 99%, 97 and 88%, respectively.

Regarding the log-rank tests performed on the bPFS and cPFS curves, for the Candiolo nomogram the overall tests are highly significant for both endpoints (P < 0.0001) and the tests for paired curves are all significant (P < 0.05) except the difference between low and intermediate risk for bPFS (P = 0.37) and between very-low and low risk for cPFS (P = 0.47). For D’Amico classification the overall Log-Rank tests are highly significant for both bPFS and cPFS (P = 0.004 and P = 0.001, respectively), and the tests for paired curves are all significant (P < 0.05) except the difference between low and intermediate risk for cPFS (P = 0.52).

Analyzing the concordance indexes, for the Candiolo nomogram the C indexes are 75 and 80% for bPFS and cPFS, respectively, while for the classification of D’Amico they are equal to 64 and 69% for bPFS and cPFS, respectively.

## Discussion

The external validation of the Candiolo nomogram on our series of 561 patients affected by prostate cancer and treated with radical Radiotherapy was overall successful. In fact, Harrell’s C concordance indexes are about 10% higher than the D’Amico control classification for bPFS (75% versus 64%) and cPFS (80% versus 69%).

In particular, the Candiolo nomogram can better identify patients with a high and very-high risk of relapse equal to only 15% of the total cases but subject to 48% (27/56) of biochemical relapses and 63% (19/30) of clinical progressions.

Furthermore, the Candiolo nomogram can identify a greater number of low-risk patients than the classification of D’Amico. In fact, according to the D’Amico classification 21% of patients belong to the low risk class with a bPFS of 95% and a cPFS of 99%; according to Candiolo, the sum of very-low and low risk patients is equal to 61% of total cases with an average bPFS of 95% and an average cPFS of 98%, substantially equivalent to the survivals of D’Amico low risk class.

However, while the distinction of the two high and very-high risk groups from the remaining risk classes is clear, it can be noted that the low risk curve sticks alternatively to the intermediate risk in bPFS and the very-low risk in cPFS.

To identify the factors that may have reduced the statistical significance in the external validation study, a comparison between the cases used in the training and validation cohorts, illustrated in Table 3, may be useful.

**Table 3:** Comparison between the training cohort and the validation cohort of the Candiolo nomogram

Clinical characteristics	Training cohort	Validation cohort
Sample size	2,493	561
Median Follow-up, mo	50	50
Mean age, yy	71.7	71.9
mean PSA, ng/ml	15.0	12.93
*T staging, %*		
cT1	30.5%	63%
cT2	57.5%	33%
cT3-4	12%	4%
*bGS, %*		
≤ 6	48%	39%
3 + 4	22%	31%
4 + 3	11.5%	12%
8	12%	10%
9–10	6.5%	8%
Biopsy cores sampled, mean no	10.3	11.0
%PC, mean %	44.3%	41.3%
*D’Amico risk class, %*		
Low	21.5%	21%
Intermediate	32%	40%
High	46.5%	39%
*Candiolo risk class, %*		
Very-low	21%	24%
Low	31%	37%
Intermediate	28%	24%
High	13%	10%
Very-high	7%	5%
*RT dose, ED2Gy*		
Mean (SD)	75.5 (3.0)	77.7 (2.4)
Median (min–max)	76.0 (67.1–81.1)	78.0 (72–82)
Exclusive RT, %	38%	24%
RT + ADT, %	62%	76%

First, an important factor for the statistical power of the study is the difference in the sample size, respectively of 2493 and 561 patients for the training and validation cohorts, respectively.

In addition, a slight difference can be noted between the two cohorts with a greater percentage of very-low or low risk cases according to Candiolo nomogram in the validation cohort compared to the training one (61% versus 52%, respectively) and a lower percentage of high and very-high risk cases (15% versus 20%, respectively).

Besides, the validation series was treated with slightly higher and more homogeneous radiotherapy doses than the training one (mean dose of 77.7 versus 75.5 Gy and SD of 2.4 versus 3.0 Gy, respectively).

Regarding therapy, a second important factor of discrepancy between the two series appears: in fact, the validation patients were treated in a greater percentage with a schedule combining Radiotherapy and ADT (76% versus 62% in the training series). Furthermore, in the validation series, ADT, when administered, was heavy both in terms of duration (13 months on average) and of the pharmacological class used (LHRH-analogue or TAB in 63% of cases). The intensive use of ADT can account for the flattening upwards of the low and intermediate risk curves of the Candiolo nomogram and of the D’Amico classification in both bPFS and cPFS graphs. So, ADT may cause a shoulder in the survival curves, early in the follow-up for bPFS (combined radiotherapy and ADT in the first 12 months of follow-up) or later in the follow-up for cPFS (rescue ADT after biochemical relapse).

The combined effect of a lower statistical power and the ADT shoulder could explain the deficit of statistical significance in the distinction between low and intermediate risks in the Candiolo nomogram (non-statistically significant differences between low and intermediate risk for bPFS and between very-low and low risk for cPFS) as well as in the D’Amico control classification (non-statistically significant difference between low and intermediate risk for cPFS).

Another question to be explored is also how much the D’Amico classification published the first time in 1999 [3] can currently be considered the golden standard classification with which to compare; in fact, even if the classification of D’Amico is still widely used by Radiation Oncologists and Urologists for its simplicity and robustness, the newest classification system is the NCCN classification of 2018 [12–15]. The NCCN classification combines PSA at diagnosis, TNM clinical staging, bGS, number of positive cores at biopsy, percentage of cancer inside each core, and PSA density into a classification of six risk classes, i.e. very-low, low, intermediate favorable, intermediate unfavorable, high, and very-high risk (excluding regional class N1 and metastatic class M1).

Comparing the Candiolo nomogram to the NCCN classification, we can see several affinities: the subdivision of the Gleason Score into 5 risk classes (≤ 6, 3 + 4, 4 + 3, 8 and 9–10); the attempt to integrate the information concerning the extension of the tumor at biopsy (percentage or number of positive cores) within the risk groups; and a greater number of risk groups than the traditional three classes of D’Amico.

Among the advantages of the Candiolo nomogram are the following: it takes into account a smaller number of total factors than the NCCN classification; overall it is a simpler classification and it can be applied quickly using the nomogram scores; uses the prognostic factor age (easily available); in the very-low risk, it does not require data on the percentage of cancer within each core or on the prostate volume for the calculation of the PSA density (needing a detailed histological examination and an ultrasound exam of the prostate with an estimation of the prostate volume); besides, it classifies in the very-low risk class a wider number of patients, including some with a bGS of 3 + 4 and even a few with 4 + 3, that may be eligible for active surveillance [16].

Among the advantages of the NCCN classification are the following: it provides for a greater division into 6 risk groups compared to the 5 of the Candiolo nomogram, in particular by dividing the intermediate risk into intermediate favorable and intermediate unfavorable, useful for the definition of patients suffering from intermediate risk in which a prophylactic pelvic irradiation of the lymph node drainage stations may be recommended; furthermore, it considers PSA density as an additional factor for the classification of very-low risk tumors, worthy of watchful waiting.

It should also be noted that, given the importance of the extension of cancer at biopsy as a prognostic factor in both the Candiolo and NCCN classifications, a potential danger of reduced applicability of the two classifications could arise in the future linked to the modality of the biopsy procedure. In fact, nowadays most prostate cancers are biopsied under ultrasound guidance by sampling the whole prostate (10–12 cores, sampled in the right and left apex, middle gland, and base): the result is therefore a reliable estimate of the extent of cancer with respect to the total prostate volume. A few cancers are already biopsied by targeting the suspect nodule visualized by multi-parametric MRI under the guidance of the same MRI or by ultrasound guidance with image fusion between multi-parametric MRI and ultrasound [17, 18]: the cores sampled with these targeted procedures are often positive and therefore overestimate, sometimes widely, the extent of the cancer compared to the whole prostate. If MRI-guided targeted biopsy techniques will spread widely, the data on the extension of cancer at biopsy would therefore be overestimated, or even worthless for prognostic purposes, limiting the accuracy or even the applicability of both classifications. A possible solution would be to use data strictly associated with the number of positive cores, but less or not affected at all by a targeted biopsy, such as the percentage of tumor inside each core (even in the presence of positive targeted cores a small nodule will still contain healthy tissue entering and exiting the nodule within the core) [19] or a radiological volumetric estimate of the extent of the cancer (for example by MRI). However, this approach would require ancillary studies to confirm a high correlation between the percentage or number of positive cores and the percentage of tumor inside each core or, even better, the radiological volume of prostate cancer, so that these parameters can reliably substitute the data on positive cores.

It should also be considered that patients in our study were staged mostly with CT, MRI and bone scan, while choline-CT-PET was not used, nor the more modern PSMA tracer. This might have lowered the ability to detect nodal and bone metastases at diagnosis in the high risk population.

Finally, it must always be taken into consideration that the choice of a different pre-treatment classification system (D’Amico, Candiolo, NCCN) can have important consequences on the therapeutic choices, such as dose escalation on prostate cancer [20], irradiation of the seminal vesicles [21, 22], prophylactic pelvic nodal irradiation [23], use of a combined treatment including Radiotherapy and short-term or long-term ADT [24, 25].

## Conclusions

The external validation of the Candiolo nomogram on a series of 561 patients affected by prostate cancer and treated with radical Radiotherapy was overall successful. In fact, Harrell’s C concordance indexes are about 10% higher than the D’Amico control classification for biochemical Progression Free Survival (75% versus 64%, respectively) and for clinical Progression Free Survival (80% versus 69%, respectively).

In particular, the Candiolo nomogram can identify a greater number of patients with low and very-low risk prostate cancer (61% versus 21% according to D’Amico classification) and it better picks out patients with high and very-high risk of relapse, equal to only 15% of the total cases but subject to 48% (27/56) of biochemical relapses and 63% (19/30) of clinical progressions.

Besides, we recommend the development of a nomogram integrating pre-treatment diagnostic risk factors with therapeutic information (like RT dose, ADT and rectal-bladder preparation protocol for radiotherapy set-up) [26, 27] and we also advise the development of follow-up programs customized to the patient’s risk of relapse.

Therefore, the Candiolo nomogram has overcome the external validation with respect to the traditional classification of D’Amico and its clinical use is justified in prostate cancer patients for risk class assessment before radical Radiotherapy.

## Data Availability

The datasets used and/or analyzed during the current study are available from the corresponding author on reasonable request.

## Abbreviations

bGS: Biopsy Gleason Score
%PC: Percentage of biopsy positive cores
EBRT: External-beam radiotherapy
bPFS: Biochemical progression free survival
cPFS: Clinical progression free survival
ADT: Androgen deprivation therapy
DRE: Digital rectal examination
TRUS: Trans-rectal ultrasound
3DCRT: 3D conformational radiotherapy
IMRT: Intensity-modulated radiotherapy
IGRT: Image-guided radiotherapy
CTV,: Clinical target volume
TAB: Total androgenic block
ED2Gy: Equivalent dose at 2 Gy per fraction
